# Acute cerebellitis in varicella: a ten year case series and systematic review of the literature

**DOI:** 10.1186/1824-7288-40-57

**Published:** 2014-06-19

**Authors:** Elena Bozzola, Mauro Bozzola, Alberto Eugenio Tozzi, Valeria Calcaterra, Daniela Longo, Andrzej Krzystofiak, Alberto Villani

**Affiliations:** 1 Department of Pediatric, Bambino Gesù Children’s Hospital, Pediatric and Infectious Diseases Unit, IRCCS, Rome, Italy; 2 IRCCS Policlinico San Matteo Pavia, University of Pavia, Pavia, Italy; 3 Epidemiology Unit, Bambino Gesù Children’s Hospital, IRCCS, Rome, Italy; 4 Department of Imaging, Neuroradiology Unit, Bambino Gesù Children’s Hospital, IRCCS, Rome, Italy

**Keywords:** Varicella, Cerebellitis, Children

## Abstract

**Background:**

Acute cerebellitis (AC) is the most common neurological complication of varicella. Nevertheless, it has been scarcely studied. The objective of this study were to asses the occurrence of AC among children hospitalized for varicella and to analyze its specific clinical picture and outcome.

**Methods:**

We retrospectively reviewed the medical records of children admitted to the hospital for varicella between 1^st^ October 2003 and 1^st^ June 2013 and we compared our results with literature. Children were all unvaccinated for varicella.

**Results:**

In our case series, AC was found out in 48 out of 457 patients (10.5%). The highest frequency of AC was observed in children from 1 to 5 years of age (60.9%). The most characteristic symptom of AC was a broad-based gait disturbance that progressed gradually over the course of a few days (95.8%). Other common symptoms included slurred speech (37.5%), vomiting (31.25%), headache (29.16%), dysmetry (25%) and tremor (22.91%). After a long hospitalization (median of 11 days), all but one children were dismissed without invalidating sequelae.

**Conclusions:**

Data from this study may help to better address the problem of varicella cerebellar complications in hospitalized children and to monitor changes over time caused by an increase in vaccination coverage.

## Introduction

Varicella is an acute, exanthematous, and highly infectious disease affecting virtually every child in the absence of vaccination programs. Varicella has mostly an uncomplicated course in early childhood. Nevertheless, it may result in severe complications [1]. A common measure of varicella complications is derived from the hospitalization of patients. In a 10-year study of varicella hospitalizations, we previously assessed the occurence of central nervous system complications, comparing our results with those from the literature. Among neurological complications, acute cerebellitis (AC) was the most frequent manifestation [2]. However, the occurrence of AC complicating varicella in hospitalized children and its detailed clinical characteristics are unknown.

### Aim of the study

The objectives of this study were to assess the occurrence of AC out of neurological complications of varicella in hospitalized children and to define its specific clinical picture and final outcome.

## Materials and methods

**Figure 1:**
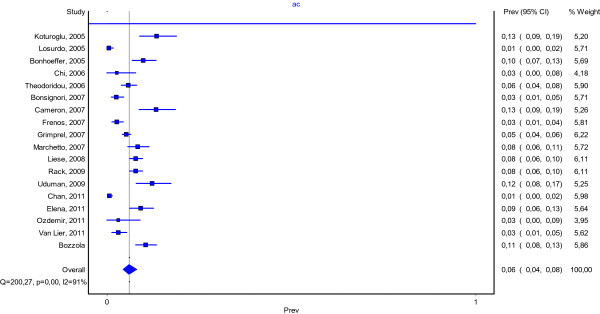
Acute cerebellitis in varicella.

We retrospectively reviewed the medical records of children admitted to the Bambino Gesù Hospital, Rome, Italy (OPBG), for varicella between 1^st^ October 2003 and 1^st^ June 2013. Patients over 18 years of age as well as children with immunodeficiency disorders were excluded. According to the literature, the diagnosis of varicella is based on clinical evidence of characteristic skin lesions in varying stages of development and resolution. We defined a neurological complication as an unfavorable neurological evolution occurring within three weeks of varicella onset [3]. AC was defined by clinical findings (ataxia, unsteady gait or fine motor movement, trembling of the head and trunk in an upright position and the extremities when attempting to move against gravity) [4-6]. The diagnosis was clinical as the onset of ataxia following the appearance of typical chickenpox rash requires no further diagnostic testing [7]. In three patients, a lumbar puncture was performed, revealing clear cerebrospinal fluid with 1–2 WBC/mm^3^ and no microorganisms on gram stain. Real time PCR for varicella zoster virus was positive in all cerebrospinal fluid samples. Magnetic resonance imaging (MRI) was performed in eighteen patients and computed tomography (CT) in two. To measure the frequency of cerebellar complication, a MEDLINE search was made using the keywords “Chickenpox/complications” as MESH term. The results were limited to publications written in English, concerning the pediatric age (0–18 years) and published during the period between June 2003 and June 2013. We scanned the references of all included articles for additional studies. Inclusion criteria for our systemic review of the literature were: (1) cases of varicella in the pediatric age; (2) reported case definition for cerebellar complications; (3) cerebellar complications reported with clear numerators and denominators. Exclusion criteria were: (1) reports not referring the exact number of cerebellar complications in varicella in a well defined study period; (2) reports limited to immunocompromised children or to children affected by underlying diseases; (3) single case reports; (4) reports limited to adults; (5) studies reporting data on both adults and children, not analyzable separately; (6) reports including both hospitalized and non hospitalized children. From each article analyzed we extrapolated the total number of varicella cases, the number of cases with neurological complications and the number of cases with cerebellar involvement. We examined a total of 249 manuscripts resulting from our MEDLINE search or from additional references found during the review. Out of these, 17 studies were eligible for inclusion in the meta-analysis and were analyzed to estimate a pooled rate of cerebellar complications in the pediatric age [1,4,6,8-21]. We excluded 233 articles for the following reasons: (1) not reporting the exact number of neurological complications in varicella in a well defined study period (211); (2) not reporting the exact number of cerebellar complications in varicella in a well defined study period (6); (3) single case reports on AC (1); (4) case reports on neurological complications different from AC (11); (5) reporting data on both adults and children, not analyzable separately (2); 6) inclusion criteria were too strict (1) (one report included only children younger than one year). Finally, two studies referred to the same group of patients. We pooled the estimates of rates and their 95% confidence intervals (CI) by using standard meta-analytic techniques. Data were analyzed using Metanalysis 3 and a pooled estimate of the occurrence of varicella complications was calculated by using a random-effects model with inverse-variance weighting using the Der-Simonian and Laird method. Statistical heterogeneity was measured by using the chi square test for heterogeneity. We used the Chi square test or the Fisher exact test for comparing proportions. We used the Standard T test for comparing continuous variables. The pooled frequency of varicella related cerebellar complications is reported in Figure 1. There was substantial heterogeneity among the studied included in the analysis (p < 0.001).

## Results

### Case series

**Figure 2:**
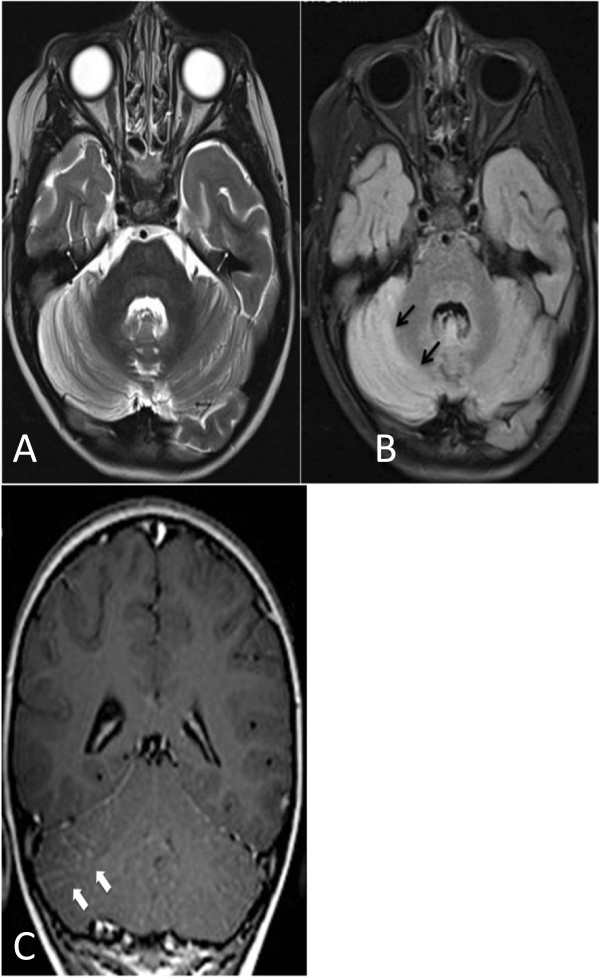
**MRI images. A**: axial T2-weighted image; **B**: axial FLAIR sequence. Diffuse high signal intensity of both cerebellar hemispheres with prevalence on the right lobe (black arrows). **C**: axial T1-weighted image after administration of paramagnetic contrast: pial disomogeneus contrast-enhancement of both cerebellar hemispheres, more evident on the right lobe (white arrows).

We reviewed 457 charts of children with varicella hospitalized during the study period. Neurological complications were identified in 92 out of 457 patients (20.13%). The mean age of patients affected by neurological complications was 5.03 years (range 2 months–15.6 years); a similar proportion of varicella cases occurred in males and females (53.4% and 46.5%, respectively). The most frequent complication among neurological complications was AC (52.17%), reported in 48 out of 457 patients (10.5%). The mean age of patients affected by cerebellar complications was 5.5 years (range 1.5–15.6 years). The highest frequency of cases was observed in children aged 1 to 5 years (60.9%). Children aged 5–10 years were 34.1% and those over 10 years were 5% of the cases. An identical proportion of cerebellar complications was observed in males and females (50%). They were all unvaccinated for varicella. The most characteristic symptom of AC was a broad-based gait disturbance that progressed gradually over the course of the first two days recovery (95.8%). Other common symptoms included slurred speech (37.5%), vomiting (31.25%), headache (29.16%) and tremor (22.91%). Poor coordination of finger-to-nose movements (dysmetry) was observed in 25% of cases. In a few cases, irritability (8.33%) and nystagmus (4.16%) were noted. Tone, deep tendon reflexes and plantar responses were normal in all patients. The mean time of onset for cerebellar symptoms was 7.48 days before hospitalization (range 1–21 days). In four patients neurological symptoms developed in the first two days. Only two children presented with late-onset complications after 14 days and within 21 days of varicella exanthema appearance. In no case did cerebellar symptoms precede skin lesions. The median length of the hospital stay was 11 days (range 2–23 days). Treatment was decided independently case by case. Forty-five patients (93.75%) were treated with intravenous acyclovir for five days (30 mg/kg, daily). In three cases, antiviral therapy was not prescribed as these children previously received oral acyclovir at home for seven days from the onset of varicella. Moreover, 16 patients (33.33%) received intravenous steroids (dexamethasone 0.5 mg/Kg/daily) due to disease severity, which required a prolonged hospitalization. Data concerning both children treated with steroids and those untreated are reported in Table 1. CT was normal in all patients; MRI showed a hyperintense signal of the cerebellar gray matter in T2-weighted sequences in five cases. (Figure 2) After discharge, the children hospitalized with AC returned for follow-up visits in order to evaluate sequelae as he still had non intentional tremors at 2 months follow up. The mean time of follow up was 3.5 months (range from 7 days to 8 months). Only one patient presented invalidating sequelae. No cases of developmental retardation, dysarthria, hemiparesis, epilepsy, blindness, deafness or coordination disorders were reported.

**Table 1:** Clinical characteristics of patients treated with steroids and of those untreated

	**Patients treated with steroids**	**Patients untreated with steroids**	
Number of patients	16	28	
Sex (female/male)	43.7%/56.3%	53.5%/46.5%	p = 0.530
Age (years)	6.4	5.1	p = 0.196
Time between onset of varicella to hospitalization (days)	6	11	p < 0.001
Length of hospitalization (days)	16	7.9	p < 0.001
Ataxia	100%	85.7%	p = 0.279
Slurred speech	50%	35.7%	p = 0.353
Vomiting	43.7%	28.5%	p = 0.306
Headache	43.7%	25%	p = 0.197
Dysmetry	43.7%	17.8%	p = 0.085
Tremor	31.2%	21.4%	p = 0.492
Irritability	12.5%	7.1%	p = 0.163
Nystagmus	6.2%	3.5%	p = 1

## Discussion

AC, the most common neurological complication of varicella, occurs about once in 4000 varicella cases among children. (5) Nevertheless, it has been scarcely studied and it is still debated whether it has a postinfectious, immunologic pathogenesis or a primarily infectious origin [22-25]. In our case series, the proportion of AC out of the total varicella cases was 10.5%. Reviewing the occurence of varicella AC complications in scientific published reports, the proportion of complications is slightly higher than those described by other authors, as shown by the metanalysis. In fact, in the literature revised, AC complication occurence lies within the CI of 4-8% (confidence ranges from 0% to 19%). (Figure 1) Varicella immunization has been recommended in only a few Italian Regions since 2003 and coverage has remained very low in the other Italian Regions, including the one where the majority of patients included in this study came from. Therefore, the results that we obtained reflect a scenario not affected by immunization. Indeed, none of the patients included had been vaccinated against varicella. In our case series, the median age of children affected by AC was about 5.5 years. In the literature, the median age of children affected by AC was 4.29 years [1,5,9,14,16]. Moreover, in our case series, children with AC were significantly older than the other children hospitalized for varicella (median age 3.2 years; P-values <0.001). They were also older than children affected by other neurological complications (median age 5.06 years). Instead in a previous report in which children affected by AC (median age 4.4) were younger than all other children with neurologic complications (median age 5.4 years). (1) The mean time of onset for cerebellar symptoms was 7.48 days before hospitalization (range 1–21 days). This is in line with the literature, in which the median time between the onset of exanthema and hospital admission was 7 days. (1) Children remained a median of 11.11 days (range 2–23 days) in hospital. The hospitalization of our patients was longer than those reported in the literature (6.72 days) [1,9,16]. This may be due to the fact that four children had a complicated disease course, which required steroids for more than 14 days. At admission, ataxia was the most frequent symptom, with wide-based gait (95.80%). Neurological presentation was also often characterized by dysmetry and difficult speech. Vomiting and cephalea were frequent, while nystagmus or other involuntary eye movements were rare. Moreover, non cerebellar symptoms, such as headache, were frequently referred by patients. In the literature reviewed, we did not find any description of the clinical presentation for cerebellitis, which would have been useful to compare with our data [5]. In our case-series, diagnosis was made based on patient history of varicella infection and physical examination. In fact, the onset of ataxia following the appearance of a typical chickenpox rash requires no further diagnostic testing. (7) In five cases, a MRI showed hyperintense signal of the cerebellar gray matter in T2-weighted sequences, which is suggestive for acute cerebellitis. Anyway, the result of these tests did not change the treatment. Brain imaging is not necessary for most cases of AC. When it is performed, MRI is vastly superior to CT. In fact, CT is of limited value given the difficulty of imaging the posterior fossa with this modality. Moreover, when obtained, CT is most often normal [26]. At MRI, bilateral diffuse abnormalities of the cerebellar hemispheres are the most common imaging presentations but are not patognomonic and with a no evident prognostic value [27]. The role of antiviral therapy is controversial. Some authors reported that acyclovir is indicated because of disease severity, while others did not recommend it, based on the strength of evidence regarding autoimmune pathogenesis [22-25,28,29]. The real utility of steroids is controversial as well [28]. As international guidelines do not clearly establish whether immunocompetent children with cerebellitis should receive intravenous acyclovir and/or steroids, we decided case by case, based on clinical severity. In our case-series, forty-five patients (93.75%) were treated with intravenous acyclovir, and 16 (33.33%) received intravenous steroids. We prescribed antiviral therapy in order to reduce disease severity; in fact, in a recent article on varicella, treatment with antivirals was considered mandatory not only for patients at risk for severe disease, but also for any subject with varicella-zoster virus infection with virally mediated complications, such as AC [29]. In the revised literature, we found just two papers reporting the frequency of antiviral therapy and only one on the steroids [1,4]. Out of 67 children, Rack et al. treated 46% with acyclovir [1]. Marchetto et al. used antiviral and steroidal therapy in respectively 68.9% and 79% of the 29 enrolled patients [4]. Finally, as well as in our case-series, other authors generally did not refer invalidating problems at the follow-up [1,14].

## Conclusion

AC is frequent during varicella in childhood and is associated with prolonged hospitalization. Neurological presentation is mostly characterized by ataxia, difficult speech, vomiting, headache and dysmetry. Data from this study may help to better address the problem of varicella cerebellar complications in hospitalized children and to monitor changes over time caused by an increase in vaccination coverage.

## Competing interests

The authors have no competing interests to declare.

## Authors’ contribution

BE provided medical assistance to the patient and collected medical information, BM and CV revised the literature, LD supervised the neuroradiological examination included in the case report, TAE supervised the examination examination included in the case report, KA was involved in the clinical follow-up of the patient, VA supervised the patient treatment plan. All authors read and approved the final manuscript.
